# Dose and Dose Rate-Dependent Effects of Low-Dose Irradiation on Inflammatory Parameters in ApoE-Deficient and Wild Type Mice

**DOI:** 10.3390/cells10113251

**Published:** 2021-11-20

**Authors:** Annegret Glasow, Ina Patties, Nicholas D. Priest, Ronald E. J. Mitchel, Guido Hildebrandt, Katrin Manda

**Affiliations:** 1 Department of Radiation Oncology, University of Leipzig, 04103 Leipzig, Germany; ina.patties@medizin.uni-leipzig.de; 2 Département de Chimie, Université Laval, Québec, QC G1V 0A6, Canada; prof.nick.priest@gmail.com; 3 Radiological Protection Research and Instrumentation Branch, Canadian Nuclear Laboratories (Retired), Chalk River, ON K0J 1J0, Canada; mitchelronliz@gmail.com; 4 Department of Radiation Oncology, University of Rostock, 18059 Rostock, Germany; guido.hildebrandt@uni-rostock.de (G.H.); katrin.manda@uni-rostock.de (K.M.)

**Keywords:** low dose, irradiation, ApoE, inflammation, endothelial, cytokine, adhesion, dose rate

## Abstract

Anti-inflammatory low-dose therapy is well established, whereas the immunomodulatory impact of doses below 0.1 Gy is much less clear. In this study, we investigated dose, dose rate and time-dependent effects in a dose range of 0.005 to 2 Gy on immune parameters after whole body irradiation (IR) using a pro-inflammatory (ApoE−/−) and a wild type mouse model. Long-term effects on spleen function (proliferation, monocyte expression) were analyzed 3 months, and short-term effects on immune plasma parameters (IL6, IL10, IL12p70, KC, MCP1, INFγ, TGFβ, fibrinogen, sICAM, sVCAM, sE-selectin/CD62) were analyzed 1, 7 and 28 days after Co60 γ-irradiation (IR) at low dose rate (LDR, 0.001 Gy/day) and at high dose rate (HDR). In vitro measurements of murine monocyte (WEHI-274.1) adhesion and cytokine release (KC, MCP1, IL6, TGFβ) after low-dose IR (150 kV X-ray unit) of murine endothelial cell (EC) lines (H5V, mlEND1, bEND3) supplement the data. RT-PCR revealed significant reduction of Ki67 and CD68 expression in the spleen of ApoE−/− mice after 0.025 to 2 Gy exposure at HDR, but only after 2 Gy at LDR. Plasma levels in wild type mice, showed non-linear time-dependent induction of proinflammatory cytokines and reduction of TGFβ at doses as low as 0.005 Gy at both dose rates, whereas sICAM and fibrinogen levels changed in a dose rate-specific manner. In ApoE−/− mice, levels of sICAM increased and fibrinogen decreased at both dose rates, whereas TGFβ increased mainly at HDR. Non-irradiated plasma samples revealed significant age-related enhancement of cytokines and adhesion molecules except for sICAM. In vitro data indicate that endothelial cells may contribute to systemic IR effects and confirm changes of adhesion properties suggested by altered sICAM plasma levels. The differential immunomodulatory effects shown here provide insights in inflammatory changes occurring at doses far below standard anti-inflammatory therapy and are of particular importance after diagnostic and chronic environmental exposures.

## 1. Introduction

Radiotherapy is a classic pillar in tumor therapy and has been used successfully for many decades to treat cancer patients. In addition to induction of tumor cell death, high doses of ionizing radiation (IR) are able to induce pro-inflammatory processes in the immune system [1]. After low doses of IR, however, anti-inflammatory and analgesic effects are observed and clinically utilized [2,3,4]. Low-dose radiation therapy, in which 5 to 10% of the total doses of high-dose IR are used, is practiced meanwhile in many European countries [5,6]. A variety of chronic inflammatory and painful joint diseases such as calcaneal spur [7] or osteoarthritis [8] as well as humeroscapular periarthritis [9] is treated by low-dose IR.

The radiobiological mechanisms behind, however, are only partially understood. Based on the results of in vitro experiments, a number of mechanisms and factors that play a role in the anti-inflammatory effects of low-dose IR have been characterized; in vivo models revealed improvements of clinical symptoms and various parameters [10]. A multi-level relationship between low-dose IR and inflammatory cascades includes on the one hand the alteration of the inflammatory properties of leukocytes, macrophages, fibroblasts and endothelial cells and on the other hand the modulated secretion of cytokines, chemokines and growth factors [11,12].

Irradiation of activated macrophages with low doses from 0.3–1.25 Gy results in decreased expressions of nitric oxide (NO) production and of the inducible nitric oxide synthase (iNOS) [13]. Additionally, low doses (<1 Gy) of IR reduce the oxidative burst, measured by the release of reactive oxygen species (ROS) and superoxide production, in activated macrophages [14]. These observations are mechanistically linked to the anti-inflammatory effects of low-dose IR. Other studies show a reduced adhesion of peripheral blood mononuclear cells (PBMCs) to endothelial cells after irradiation doses of 0.3–0.7 Gy along with an enhanced expression of TGFβ and a decreased E-selectin expression [12,15,16,17]. Cytokines and chemokines contribute to all aspects of inflammatory reactions in different ways and interact with various cells of the immune system [18,19,20]. Immune modulatory effects are seen here already at very low doses. We found that IR at doses as low as 0.01–0.05 Gy causes non-linear, dose-dependent alterations of inflammatory cytokines in murine endothelial cell lines and human umbilical vein endothelial cells dependent on culture conditions (2D/3D) and activation by TNF-α [21,22].

Radiation in this dose range is particularly relevant for people with exposure in occupational environments such as employees in the medical field, air traffic and in the nuclear industry; for people in exposed environmental areas (e.g., residents of regions with high radon content) and also for patients requiring diagnostic imaging such as X-ray, CT or mammography [23,24,25,26,27]. However, knowledge about the effects of very low doses of radiation is still very limited. Therefore, it is important to research the effects of low-dose IR (<0.1 Gy) further, especially with regard to preexisting imbalances of the immune system, which may result in differential responses.

Previously we could show that low-dose IR (0.025–2 Gy) can induce pro and anti-inflammatory long-term (4–8 months after IR) effects on plasma parameters and affects the heart microvasculature in the inflammation and atherosclerosis prone ApoE−/− mouse model [28]. ApoE is a plasma cholesterol carrier protein which exerts important inhibitory functions on cellular and soluble components of the immune system [29,30]. Therefore, the ApoE knockout (ApoE−/−) model is used here to investigate low-dose IR effects in a situation of inflammation and persistent hyperactivity of the immune system along with hypercholesterinemia and atherosclerosis [31]. From above animals [28], we here analyzed the spleen, hosting wide functions on the adaptive and innate immune system, and present differential dose rate-dependent results of macrophage ratio and cell proliferation. Furthermore, we expand above investigations on immunogenic plasma parameters towards earlier time points (7–28 days after IR) and very low-dose IR (0.005–2 Gy) to observe acute responses and early-stage disease using an ApoE wild type mice in addition to the proinflammatory ApoE−/− mouse model. Complement in vitro experiments verified the role of endothelial cells in this process by analyses of monocyte adhesion and cytokine release after low-dose IR. We present effects on pro and anti-inflammatory cytokine levels (IL6, IL10, KC, IL12p70, MCP1, INFγ, TGFβ) as well as on expression of the thrombotic biomarker (fibrinogen) and adhesion molecules, involved in the activation and injury of the endothelium (VCAM, ICAM, sE-selectin).

Throughout this paper, the following definitions for radiation doses will apply: low doses: <0.1 Gy, intermediate/medium/moderate dose: 0.1–1 Gy, high dose: >1 Gy.

## 2. Materials and Methods

### 2.1. Mice, Irradiation and Analysis of Plasma Samples

ApoE−/− (B6.129P2-ApoEtml/unc/J9) female mice, homozygous null for a functional ApoE gene (in C57BL/6J) background as well as ApoE+/+ control mice were transferred from Jackson Laboratory (Bar Harbor, ME, USA) at 6 weeks of age to the Canadian Nuclear Laboratories (Chalk River, ON, Canada). Mice were provided with sterilized water ad libitum and Charles River Rodent Chow no. 5075 autoclaved normal low-fat diet. Ten mice per dose group were exposed to 60Co γ-radiation (0.005, 0.025, 0.1, 0.5 and 2.0 Gy) at 8 weeks of age. IR was performed at either low dose rate (LDR, 0.001 Gy/min, 0.1 Gy/day) from an open beam source (GammaBeam 150, Canadian Nuclear Laboratories) on unrestrained mice or at high dose rate (HDR, 0.15–0.36 Gy/min) in an enclosed irradiator (GammaCell 200, Canadian Nuclear Laboratories). All groups received their doses as single exposure except for 0.5 and 2.0 Gy LDR groups. These mice were irradiated at 0.001 Gy/min (0.1 Gy/day) for 5 days/week for 4 weeks at longest.

EDTA plasma samples (35–50 µL) were taken 7 days prior IR time point (I), and 24 h (II), 7 days (III) as well as 28 days (IV) after IR start (Charles River Canada, St. Constant, Quebec). EDTA plasma samples were sent to the University of Leipzig and stored at −80 °C until measurement.

Murine interleukins 6, 10 and 12p70, chemokine KC, macrophage chemoattractant protein-1 (MCP1), tumor necrosis factor alpha (TNF**α**) and interferon gamma (IFN) were measured by cytometric bead array (CBA) using the mouse inflammation kit (BD Biosciences) on the BD FACSArray System as described previously [28]. Soluble adhesion molecules: intercellular adhesion molecule (sICAM), vascular adhesion molecule (sVCAM), endothelial leukocyte adhesion molecule 1 (sE-selectin, CD62); tumor growth factor beta (TGF**β**1) and fibrinogen were measured by ELISA (sICAM: Thermo Scientific; sVCAM, sE-selectin, TGF**β**1: R&D Systems; fibrinogen: BioCat) and analyzed using ANTHOS zenyth 340r reader (Anthos Mikrosysteme GmbH, Krefeld, Germany).

### 2.2. Expression of Immune Parameters in Spleen

Spleen samples from previously irradiated ApoE−/− mice, detailed and published in [28] were analyzed by realtime RT-PCR for expression of CD68 and Ki67. Shortly, mice were irradiated using 0.025 to 2 Gy, at LDR or HDR. Three months after IR, mice were euthanized and different organs including spleen isolated and stored at minus 80 °C until further use. RNA from four mice per IR dose and specific dose rate was isolated by chloroform/phenol precipitation (RNAzolRT, Sigma, St. Louis, MO, USA). RNA (1.5 µg per sample) was transcribed into cDNA using random N6 primers, which resulted in higher Ct values for Ki67 than oligo (dT) primers, and Omniscript RT kit (Qiagen, Dusseldorf, Germany). RT-PCR was conducted on a Corbett Rotor-Gene 3000 light cycler using Takyon (NoRoxProbeUNG) master mix with carry over prevention (Eurogentec, Liege, Belgium) and primers/dual labeled probes (Metabion, Steinkirchen, Germany) listed in Table 1. RT-PCR was conducted twice per sample for the house keeping genes. Standard curves were conducted for each gene in triplicates. Others have shown by analysis of 11 house keeping genes that 18S rRNA and β-2 microglobulin (mB2m) have the most constant levels of expression following exposure to ionizing radiation [32]. Therefore, they appeared to be excellent candidates for use as internal controls in RT-PCR here.

### 2.3. In Vitro Experiments

Cell culture: The murine monocyte cell line WEHI-274.1 (ECACC, Salisbury, UK) and three murine endothelial cell (EC) lines were cultivated in Dulbecco’s modified Eagle’s medium (DMEM with 4.5 g glucose, Lonza, Basel, Switzerland) with 10% heat inactivated fetal calf serum (FCS, Sigma) and penicillin/streptomycin (Lonza) as previously described [22,34]. Polyoma middle-sized T antigen-transformed heart EC line, H5V [35], was kindly provided by Dr. Annunciata Vecchi, Centro Ricerche, Instituto Clinico Humanitas, Rozzano, Italy. Lymph node EC line, mlEND1, was kindly provided by F. Rödel, Dept. of Radiotherapy and Oncology, University Hospital Frankfurt, Frankfurt am Main, Germany, and cerebral cortex endothelial bEND3 (ATCC^®^ CRL-2299TM) was obtained from ATCC [36].

Cytokine detection and irradiation: Endothelial cells were seeded in 24-well plates (100,000 cells/0.5 mL medium/well). Medium was replaced by 0.2 mL serum-free medium (SFM) with HEPES (10 mM) 24 h later. After a two-hour rest, recombinant murine TNFα (5 ng/mL, R&D Systems) was added and, if not otherwise noted, IR followed immediately with single shot of 0.05, 0.1, 0.5, 1, 2 and 5 Gy using a 150-kV X-ray unit (Darpac 150 MC, 0.81 Gy/min). Sham-irradiated control cells (0 Gy) were treated equally. Supernatants were collected 24 h after IR and stored at −80 °C until analysis. IL6, KC, MCP1 were measured by specific CBA flex sets (BD Biosciences) at BD FACSarray, and TGF**β** (MC100B, R&D Systems) by ELISA at 450/690 nm (Spectrafluor plus, TECAN).

Adhesion assay: ECs (80,000 in 0.5 mL per chamber) were seeded in 4-well glass chamber slides (Nunc, Cat. 154526) and incubated overnight reaching confluency. Medium was replaced by SFM with 10 mM HEPES and after 2 h rest, TNFα (5 ng/mL) was added and cells were irradiated as described above followed by 24 h rest in the incubator.

Monocytes (WEHI-274.1) were added to the confluent monolayer of ECs (400,000 monocytes/chamber). After incubation for 1 h at 37 °C, chambers were removed and slides were cautiously swiveled three times with SFM/HEPES. For fluorescence staining, cells were fixed with 95% ethanol for 15 min, rinsed twice with PBS and nonspecific binding was blocked for 20 min by 10% donkey serum in PBS. Cells were incubated for 1 h with primary antibody goat anti-mouse CD45 (1:600, R&D Systems, Minneapolis, MN, USA), washed three times in PBS, followed by 45 min incubation with secondary antibody (donkey anti-goat Cy5 (1:400, Jackson ImmunoResearch Laboratories, Inc., West Grove, PA, USA). After three washes with PBS and counterstaining with DAPI (0.5 µg/mL A.dest) cells were embedded with Mowiol 4-88/DABCO (Roth). Adherent monocytes (Cy5+, Dapi+) were counted by fluorescence microscopy in fields of view containing averaged 2500 ECs (Cy5−, Dapi+) per sample.

### 2.4. Statistical Analysis

All data are presented as means ± standard error of mean (SEM) if not otherwise written. N is defined in vivo as the number of mice; in vitro as the number of independent experiments per cell line, if not otherwise described. Statistical significance was assessed by Student’s *t*-test on log data if normal distribution was not fulfilled (*, *p* ≤ 0.05; **, *p* ≤ 0.01; ***, *p* ≤ 0.001). For multiple comparisons in Figure 2, ANOVA analysis followed by Dunnett’s two-sided post hoc test has been conducted.

## 3. Results

### 3.1. In Vivo Effects of Irradiation on the Spleen

**Figure 1:**
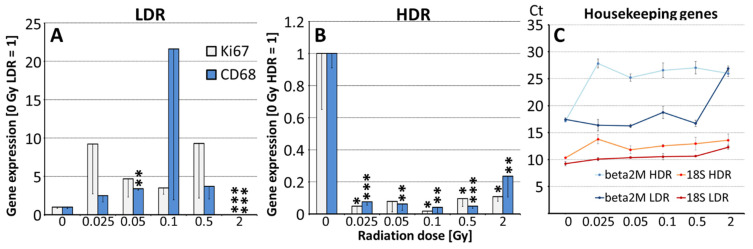
IR dose-dependent expression of Ki67 and CD68 in the spleen of ApoE−/− mice, *n* = 4. (**A**,**B**) Relative expression levels of CD68 and Ki67 normalized on 18S rRNA in the spleen, mean-SEM, 3 months after IR at low dose rate (LDR) or high dose rate (HDR) are shown. Significant changes are indicated compared to 0 Gy control (=1) by asterisks (*, *p* ≤ 0.05; **, *p* ≤ 0.01; ***, *p* ≤ 0.001). (**C**) Mean Cycle threshold (Ct) values of house keeping genes, β2M and 18 S, are presented for all samples, mean ± SEM.

Dose and dose rate-dependent effects of IR were analyzed in the spleen of irradiated ApoE−/− mice by RT-PCR, Figure 1A–C. We found differential responses after low dose rate (LDR) versus high dose rate (HDR) IR with regard to expression of the macrophage marker, CD68 (*p* ≤ 0.001) and to the proliferation marker, Ki67 (*p* ≤ 0.01). LDR-irradiated animals: Minor enhances of CD68, significant only at 0.05 Gy, and a trend to enhanced Ki67 expression are seen in the spleen at low doses. IR at 2 Gy (high dose control) significantly reduced expression levels of both markers, Figure 1A. HDR-irradiated animals: Strong significant decreases of both markers are seen already at low doses starting from 0.025 Gy and remain like this at higher doses (0.5 and 2 Gy), Figure 1B. Expression of β2M mRNA appeared to be regulated by IR, Figure 1C, therefore data were normalized on 18S rRNA whose levels remained nearly unchanged.

### 3.2. Irradiation-Induced Effects on Plasma Parameters in Mice

**Figure 2:**
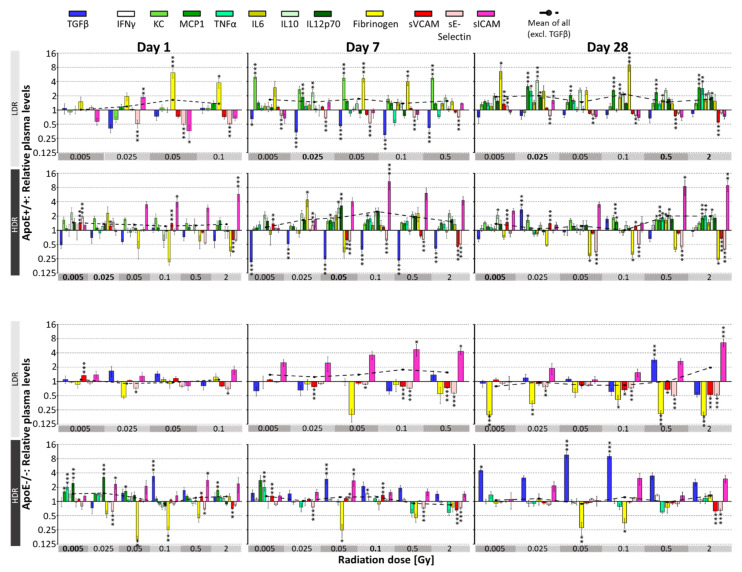
IR dose-dependent effects on plasma parameters in mice, *n* = 10. Relative plasma levels on day 1, 7, 28 after IR are presented in wild type ApoE+/+ and ApoE−/− mice after IR at low dose rate (LDR) and high dose rate (HDR). Values are normalized on individual levels 7 days before IR and are presented relative to 0 Gy control of the corresponding time point (=1). Significant changes compared to 0 Gy group at the corresponding time point are indicated by asterisks (*, *p* ≤ 0.05; **, *p* ≤ 0.01; ***, *p* ≤ 0.001). Plasma levels for dose 0.5 and 2 Gy LDR, day 1, and 2 Gy LDR, day 7 are not available because these doses could not be delivered till the indicated time points due to the low dose rate. Missing bars in ApoE−/− mice represent plasma parameters below detection limit.

In vivo effects of IR have been investigated on pro and anti-inflammatory markers reflecting mainly the endothelial response (sVCAM, sE-selectin, sICAM), cytokines (TGFβ, INFγ, TNFα, KC, MCP1, IL6, IL10, IL12p70) which can be secreted locally by ECs but may also originate from circulating immune cells and the thrombotic marker fibrinogen, produced in the liver, Figure 2. Results of subgroup-specific analysis on day 1, 7 and 28 after IR at LDR and HDR are presented, relative to sham-irradiated control (=1). Dashed line comprises the mean of all parameters considered as proinflammatory, therefore excludes TGFβ. One day after IR, this mean was significantly enhanced only after HDR treatment at 0.005 and 0.025 Gy in ApoE+/+ wild type and at 0.005 Gy in ApoE−/− mice. On day 7, significant increases were detected only in ApoE+/+ mice at 0.025 Gy LDR and 0.05 Gy HDR. Four weeks after IR (day 28) only ApoE+/+ mice at 0.005 Gy LDR showed significant enhancement of the proinflammatory mean due to the drop of fibrinogen and sVCAM/sICAM.

ANOVA analysis revealed, except for INFγ, significant, dose-dependent significant changes for all plasma markers at all three time points, compared to untreated corresponding controls, *p*. Model-specific responses were strongest for fibrinogen and TGFβ, while dose rate affected mostly TGFβ in ApoE−/− and KC, fibrinogen and sICAM levels in wild type mice.

**Figure 3:**
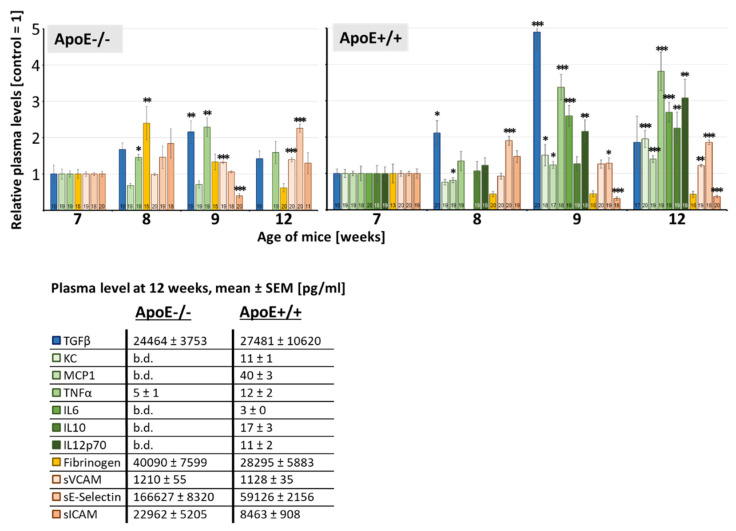
Age-dependent changes of plasma parameters in non-irradiated mice. Relative concentration of 11 plasma markers is presented in 7, 8, 9 and 12-week-old, sham-irradiated 0 Gy control ApoE−/− and ApoE+/+ mice. Significant changes compared to 7-week group (=1) are indicated by asterisks (*, *p* ≤ 0.05; **, *p* ≤ 0.01; ***, *p* ≤ 0.001). Numbers in the bars represent the number of animals (sample size). Absolute plasma values of ApoE−/− and wild type ApoE+/+ mice at 12 weeks of life are given in the table (b.d. = below detection limit).

ApoE+/+ wild type mice: Several parameters (MCP1, TNFα, IL6, IL10, IL12p70) were below detection limit, at early time points (1 day after IR). These parameters enhanced 7 and 28 days after IR but also after sham IR (0 Gy), indicating an age-dependent increase supported by findings below, Figure 3. INFγ level were below detection limit for several time points.

LDR IR enhanced fibrinogen levels immediately, reaching significance levels at 0.05 Gy, 1 day after IR. At later time points, more proinflammatory cytokines (KC, MCP1, IL6) followed this pattern even at lower doses (0.005 Gy), whereas TGFβ decreased. The soluble adhesion molecules sVCAM, and especially sE-selectin showed as well significantly reduced levels at all three time points, whereas sICAM levels varied and dropped significantly only 28 days after IR.

HDR IR showed a very different picture regarding fibrinogen and sICAM responses. SICAM levels enhanced and fibrinogen levels decreased significantly already from day 1 after IR onwards. Significance levels were reached at 0.05 Gy. Similarly to LDR, levels of proinflammatory cytokines increased and sE-selectin and TGFβ decreased, the latter effect lessening at day 28 for both dose rates.

ApoE−/− mice: The cytokine concentrations were generally slightly lower in this model than in ApoE+/+ wild type mice and mostly stayed below detection limit for the term analyzed here. Comparison of the dose rate effects revealed further differences, showing significantly decreased fibrinogen and enhanced sICAM levels at both, IR at LDR and HDR. Furthermore, IR at HDR led to enhanced TGFβ levels in contrast to the decreases found in wild-type mice. Significant decreases of sVCAM and sE-selectin resemble the findings in ApoE+/+ mice. Most changes were significant at 0.05 Gy but some at doses as low as 0.005 Gy.

### 3.3. Age-Related Effects on Plasma Parameters

Analysis of plasma parameters in sham-irradiated mice over 5 weeks revealed significant age-dependent changes, Figure 3. In ApoE−/− mice, effects were less pronounced than in wild-type mice, showing transient enhances of fibrinogen at 8 weeks and of TGFβ and MCP1 levels at 9 weeks. Endothelial proteins sVCAM and sE-selectin increased significantly at week 12. In contrast, sICAM levels remained unchanged through this period except for a transient drop at week 9.

In ApoE+/+ wild type mice, all cytokines revealed a significant age-specific rise in 12-week-old mice compared to 7-week-old mice. TGFβ showed significant transient enhancement at week 8 and 9. Fibrinogen concentrations tended to decrease. In addition, endothelial adhesion molecules sVCAM and sE-selectin enhanced, whereas sICAM levels dropped at week 9 and 12 significantly. Basal plasma levels for sE-selectin and sICAM were significantly higher in ApoE−/− versus wild type mice, age 12 weeks, *p* ≤ 0.05.

### 3.4. Irradiation of EC Lines—Effects on Cytokine Release and Monocyte Adhesion

In vitro experiments were conducted to verify if the IR-induced cytokine changes in mice could be at least partly attributed to EC response. Cytokine release and adhesion effects were measured in three murine EC cell lines (H5V, mlEND1, bEND3).

**Figure 4:**
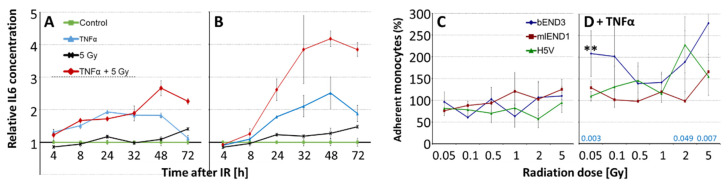
Time dependent analysis of IL6 release in mlEnd1 cells after 16 h-prestimulation (**A**) and after costimulation (**B**) with TNFα. Relative IL6 values are presented (mean of duplicates ± SEM, *n* = 1) versus untreated control (=1). Relative adhesion values of monocytes (WEHI-274.1) on H5V, mlEND1, bEND3 ECs in (**C**) nonstimulated (0 Gy control = 100%) and in (**D**) TNFα costimulated cells, (TNFα-treated 0 Gy control = 100%), *n* = 3. Asterisks shows significant enhancement vs. control in the bEND3 cell line (**, *p* ≤ 0.01). *p*-values of combined analysis of all three cell lines are shown in blue.

In preliminary time course experiments, the optimal time point for cytokine measurements and activation conditions was determined: IR (5 Gy), TNFα (5 ng/mL) and combinations of both led to a time-dependent increase of IL6 concentration in supernatants of mlEND1 cells, Figure 4. Pre-stimulation with TNFα 16 h prior IR partly overrides the effect of IR and leads to a drop of TNFα-induced IL6 levels 32 h after IR (Figure 4A). In contrast, after simultaneous/co-stimulation with TNFα the effect on IL6 release dropped only at 72 h after IR. In addition, combined (TNFα + IR) effects were much higher than after pre-stimulation, Figure 4B. Similar results were found for MCP1 and KC. Thereby, co-stimulation by TNFα/IR enhanced MCP1/KC levels 6.0/10.1-fold (4 h), 9.5/6.0-fold (8 h), 6.4/7.7-fold (24 h), 6.4/7.2-fold (48 h), and 5.0/5.8-fold (72 h) respectively, compared to untreated controls. Therefore, co-stimulation of TNFα/IR was chosen for all following experiments and measurements were conducted after 24 h.

Monocyte adhesion was investigated in all three EC cell lines 24 h after IR (0.05–5 Gy) with and without TNFα co-stimulation using the monocyte WEHI-cell line, Figure 4C,D. Thereby, TNFα did not change monocyte adhesion compared to non-stimulated cells (396 ± 164 without TNFα vs. 389 ± 168 with TNFα, adherent monocytes/1000 ECs; mean of three cell lines, *n* = 3 each). IR alone also had no significant effect (Figure 4C) but induced significantly monocyte adhesion in TNFα co-stimulated cells already at 0.05 Gy, especially in bEND3 cells. Intermediate IR doses (0.1 to 1 Gy) showed no effect, whereas higher doses (2 and 5 Gy) also enhanced TNFα-stimulated adhesion significantly compared to TNFα-treated 0 Gy control (combined analysis, three cell lines, *n* = 3 each, Figure 4D).

**Figure 5:**
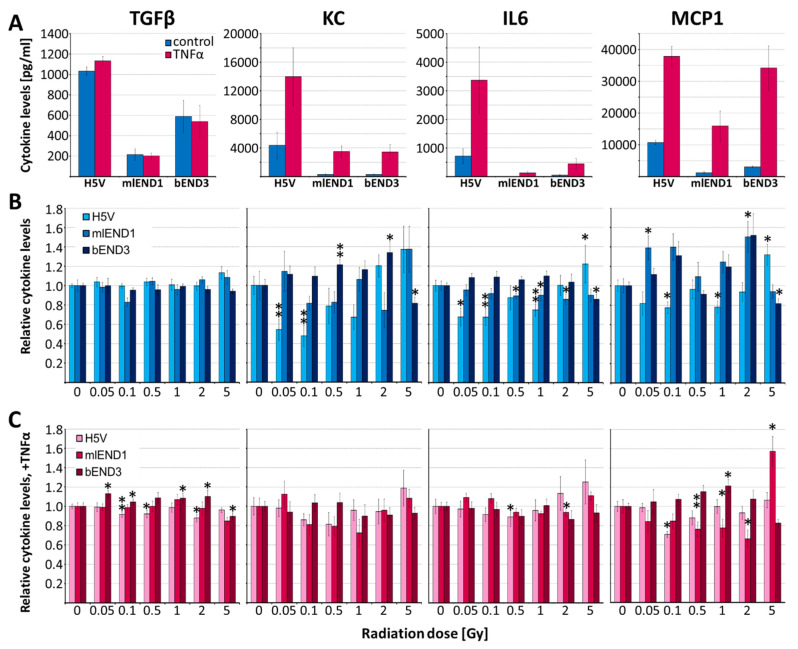
Concentration of TGFβ, KC, IL6 and MCP1 in supernatants of H5V, mlEnd1 and bEND3 endothelial cell lines. (**A**) Effect of TNFα (5 ng/mL) compared to untreated control, absolute values. (**B**,**C**) Dose-dependent effects of IR without (**B**) TNFα costimulation, relative to 0 Gy control samples (=1), and with TNFα costimulation (**C**) relative to TNFα-stimulated, 0 Gy control samples (=1) 24 h after IR. Asterisks indicate significant differences for each cell line (*, *p* ≤ 0.05; **, *p* ≤ 0.01; 3 experiments per cell line, each in quadruplicates).

IR dose-dependent cytokine release was investigated in TNFα-stimulated (resembling the inflammatory phenotype) and in unstimulated EC lines (Figure 5). TNFα (5 ng/mL) significantly enhanced the non-irradiated levels of KC, IL6 and MCP1 in all three cell lines 8.9-fold, 12.4-fold and 9.5-fold, respectively (*n* = 3, *p* ≤ 0.05), whereas TGFβ values remained unchanged (Figure 5A). IR led to differential cytokine release in the three cell lines. In general, H5V was the most and bEND3 least responsive cell line. Low-dose IR (0.1 to 0.5 Gy) reduced significantly the KC, IL6 and MCP1 release, and in TNFα-stimulated cells, the TGFβ, IL6 and MCP1 release in H5V cells. In mlEND1 unstimulated cells, MCP1 level increased slightly with non-linear dose dependency whereas IL6 level and, in TNFα-stimulated H5V cells, MCP1 level dropped after low-dose IR. In contrast, in bEND3 cells slightly increased KC, and after TNFα-costimulation, also increased TGFβ levels were found. High-dose IR (2–5 Gy) tended to enhance proinflammatory cytokine concentrations (KC, IL6 and MCP1) especially in unstimulated cells (Figure 5B,C).

## 4. Discussion

High-dose IR is known to induce endothelial cell activation and dysfunction along with inflammatory and prothrombotic changes [37,38,39]. Especially on cardiac vasculature and function, early and late effects of IR have been identified e.g., as a side effect after chest irradiation during anti-tumor therapy [40,41]. Low dose effects (<0.1 Gy), however, are less obvious, non-linear and therefore cannot be extrapolated from high doses. In ApoE−/− mice we and others have already shown that low-dose whole-body IR can exert long term immunomodulatory effects including inflammatory and fibrotic changes on the heart and vasculature as well as on plasma markers [28,42].

To get the whole picture, we performed here complementary analyses of systemic long-term (3 months) immune effects on the spleen. Furthermore, we extended this project towards investigations of acute and short-term, possibly transient low-dose IR plasma marker responses with specific attention to the delivered dose rate and the inflammatory status. Additionally, in vitro experiments were performed to investigate the role of ECs, potentially contributing to IR-induced changes of plasma markers.

### 4.1. Systemic In Vivo Responses after Whole Body Irradiation (WBI)

Long-term effects: Emphasizing the results of our previous study [28] we found differential dose rate-specific effects of low-dose IR with significantly stronger response after IR at HDR versus LDR in the spleen of ApoE−/− mice (Figure 1). Thereby, only after IR at HDR from 0.025 Gy onwards, a significant reduction of mRNA expression for Ki67, mainly representing T-cell proliferation and of CD68, representing the number of macrophages scavenging senescing erythrocytes, thrombocytes and T-cells, was detected, which is new after such a long period. Short term investigations demonstrated induction of double strand breaks and apoptosis at slightly higher doses (≥0.1 Gy, HDR) in the spleen [43]. In contrast, after IR < 0.5 Gy at LDR, we found no significant effects, which might be explained by the activation of radio-adaptive mechanisms demonstrated in mice after 1.5 Gy IR at LDR [44]. Moreover, DNA damage repair genes were found to be induced 7 days post-IR at 0.04 Gy, LDR [45].

High IR doses (2 Gy) however, reduced Ki67 and CD68 expression at both, low and high dose rate, indicating that adaptive response is limited, and cells are not able to resolve accumulation of DNA damage at high doses. Accordingly, spleen weight and size are reduced after IR with 1 Gy in mice [46] and after IR with ≥2.5 Gy e.g., for treatment of splenomegaly mostly due to lymphocyte toxicity [47,48]. Although sensitivity to IR varies, apoptosis is induced in all spleen subpopulations after IR at 2 Gy [49]. At higher doses (5 Gy) also CD68/T cell ratio may increase by lymphoid cell death and invasion of monocytes [50]. A limitation of the study is that investigation of spleen tissue could be conducted only in ApoE−/− mice as wild type mice were not part of this project.

Short-term effects: Previously we could demonstrate that whole body low-dose IR (0.025–2 Gy) exerts pro- and anti-inflammatory long-term effects in the heart and on plasma markers of ApoE−/− mice [28]. Extending those investigations towards earlier time points (1, 7 and 28 days), lower doses, and wild type mice, we could demonstrate significant cytokine, thrombotic and adhesion marker responses at doses as low as 0.005 Gy. Thereby significant model-specific as well as dose and dose rate-specific responses were revealed, Figure 2.

Enhancement of circulating soluble adhesion molecules, especially of sICAM1, has been demonstrated in inflammatory and vascular, acute or chronic diseases indicating inflammatory changes and endothelial dysfunction [51,52]. Upregulation of inflammatory adhesion molecules is also one of the earliest steps of atherosclerotic development and has been shown to be triggered by IR in cancer patients [53]. Interestingly we found that very low IR doses, from 0.005 Gy onwards, enhance sICAM levels in ApoE−/− mice and, at HDR only, also in wild type mice. These findings are in line with reports of low-dose IR induced acute upregulation of sICAM in ApoE−/− mice after thorax IR at 0.1 Gy [54]. Based on our previous investigations we assume that upregulation of ICAM after low-dose IR is only transient as it was even slightly counter-regulated after 3 months [28]. This goes along with reduced atherosclerotic lesions in ApoE−/− mice, 3 and 6 months after LDR IR exposure between 0.025 and 2 Gy [55]. Other adhesion molecules (VCAM, sE-selectin) are hardly affected and change mainly at high control dose (2 Gy). Corresponding in vitro findings (2–10 Gy), suggest that ICAM levels are most heavily regulated in IR-induced inflammatory reaction of the endothelium [56].

Most strikingly, TGFβ levels steadily increased in ApoE−/− from day 1 to 28 after HDR IR. This effect was also observed three months after IR and to a lesser extent also at LDR IR [28] and might contribute to enhanced cardiac fibrosis induced by low-dose IR in ApoE−/− but not wild type mice [42]. In wild type mice, IR after 7 days only transiently reduced TGFβ secretion underlining the differential responses towards low-dose IR depending on the inflammatory state [10].

Notably, fibrinogen levels are enhanced in wild type mice after exposure at LDR but down-regulated at HDR and at either dose rate in ApoE−/− mice. Again, this effect is transient for LDR, but for HDR ApoE−/− fibrinogen levels increase also 3 and 6 months after IR. Fibrinogen levels are elevated in systemic inflammatory processes such as cardiovascular disease, atherosclerosis and tissue injury [57,58], although the mechanisms are not completely understood. Acute phase cytokines (IL6) may increase fibrinogen [59] plasma levels and in turn, fibrinogen activates cytokine secretion in PBMCs [57]. Although high levels of TGFβ are associated with fibrosis, its anti-inflammatory properties [59] could be partly responsible for the relatively low levels of proinflammatory cytokines after low-dose IR in ApoE−/− mice. Reduced cytokine levels might then contribute to decreased fibrinogen levels there. Reversely, in wild-type mice, reduced TGFβ levels go along with increased proinflammatory cytokine response and increased fibrinogen.

Taken together, analysis of low-dose IR induced short-term response revealed significant early changes of inflammatory markers, some of them translating into long-term, partly counterregulatory effects [28] at radiation doses relevant e.g., for diagnostic imaging.

### 4.2. Age-Related Plasma Effects

Age-related changes of inflammatory markers demonstrated here in 7 to 12 week old ApoE−/− and wild type mouse models (Figure 3) supplement our findings in 2 to 8 months old ApoE−/− mice [28] and underscore the requirement of age matched controls for measurements of these plasma markers. Age-dependent enhancement of cytokines and the adhesion molecule sVCAM is similarly found in humans [60,61,62] and may reflect the age-related proinflammatory status which is thought to contribute to cardio/neurovascular diseases in the elderly. Enhanced basal levels of sE-selectin and sICAM in ApoE−/− versus wild type mice could be explained by their association with atherosclerotic diseases and enhanced cholesterol levels present in the ApoE−/− mice [28,61,63].

### 4.3. In Vitro Effects of Irradiation on Endothelial Cells

To evaluate if the changes of plasma cytokines and adhesion molecules after low-dose IR might be attributed partially to EC response, in vitro experiments on murine ECs were conducted (Figure 4 and Figure 5). Thereby, three murine EC lines were submitted to low-dose IR at HDR and selected cytokines (KC, MCP1, Il6, TGFβ) as well as monocyte adhesion were measured. The EC lines were chosen to present three different organs (brain, lymph node, heart) with the aim to generate data relevant for systemic low-dose IR enabling comparison with our in vivo data.

In accordance with our previous data in human and murine ECs [21,22,64], time-course experiments revealed that in vitro cytokine release peaked 24 to 48 h after IR (5 Gy) and/or TNFα (Figure 4). Therefore, this time point was adopted for low-dose IR analysis although responses for up to 6 days were shown by others after 10 Gy exposure [65]. Irradiation-induced immunomodulatory effects may vary depending on the activation status of the ECs [10] resulting in differential response of ApoE−/− versus wild type mice [37,38]. The pro-inflammatory EC status of ApoE−/− was mimicked in the in vitro experiments here by addition of TNFα [66]. TNFα (1–20 ng/mL) is commonly used to activate human and murine ECs in vitro inducing pro-inflammatory and pro-atherogenic chemokines like RANTES, IL8/KC and MCP1 [21,22,67,68]. Accordingly, we found significant TNFα stimulation of all three pro-inflammatory cytokines (KC, MCP1, IL6) already at 5 ng/mL and used this low concentration to simulate an inflammatory situation but to also allow for additional effects of IR (Figure 5A).

Adhesion: Intermediate IR doses are known to exert anti-inflammatory effects such as attenuation of leukocyte/endothelial adhesion e.g., by induction of TGFβ and downregulation of sE-selectin [12,17]. In contrast to IR of monocytes alone or IR of both, monocytes and ECs, highest adhesion responses are detected when ECs are irradiated [68] as was therefore done here, Figure 4. Previous in vitro experiments showed reduced adhesion after IR at 0.1 to 0.6 Gy in prestimulated murine and human EC lines. Although, this seems to be a highly dynamic process with decreased adhesion properties reported 4 and 24 h after activation of ECs with TNFα but with increased adhesion 12 h after activation [12,15]. Indeed, we found low-dose IR affects monocyte adhesion not after TNFα prestimulation for 16 h but after TNFα costimulation resulting in distinct non-linear effects typical for low doses [3]. Joined analysis of three EC lines revealed discontinuous dose response relationship with enhanced adhesion at 0.05, 2 and 5 Gy but not at intermediate doses similar to previous investigations at double TNFα concentrations [22]. Clinical relevance of these findings increases by the fact that repeated IR at intermediate doses (0.125 to 0.25 Gy) enhances ICAM1 surface exposure and endothelial adhesiveness in HUVECs [69]. Our in vitro adhesion data in the TNFα-costimulated setting correlate with enhanced sICAM levels in the plasma samples indicating non-linear changes of EC adhesion properties by IR.

Cytokine release: To evaluate a possible contribution of ECs to the IR-affected cytokine plasma levels in the above mouse models, IR effect on selected cytokines (KC, MCP1, Il6, TGFβ) was analyzed in murine ECs., Figure 5. Recently, multiplex CBA analysis identified IL8 (human equivalent for KC) in HUVECs as well as KC and MCP1 to be promising candidates which can be regulated by IR in murine EC lines [21,22]. Moreover, MCP1, IL6, TGFβ plasma levels, produced by ECs and leukocytes, change after IR also in patients [10].

Comparison of in vitro data and plasma cytokine levels, 1 day after IR at HDR reveals several similarities. In contrast to the plasma data in wild type mice (Figure 2), non-stimulated H5V and mlEND1 cells showed slightly reduced cytokines (KC, IL6, MCP-1). On the other hand, reduced MCP-1 levels in ApoE−/− mice at 0.1 Gy correspond to the findings in TNFα-costimulated H5V cells. Slightly enhanced TGFβ values are also seen in both, ApoE−/− plasma samples and bEND3 cells at 0.05 and 0.1 Gy indicating that some of the plasma cytokine changes could be partially attributed to endothelial cell response.

Differential responses of the three EC cell lines might be due to their different origin and indicate tissue specific sensitivities to IR. In summary, in vitro data confirm significant non-linear responses to low-dose IR ≥ 0.05 Gy, which differ depending on the activation status of the ECs.

## 5. Conclusions

Irradiation-induced DNA damage and ROS are known to activate intracellular stress signaling cascades linked to cytokine release, adhesion molecule expression and cell death induction.Pathogenetic mechanisms of low-dose IR involve both, the innate and the adaptive immune response exerting effects on cellular level in spleen and endothelium, and on intercellular communication through cytokines.

Long-term down-regulatory effects on immune parameters of spleen have been demonstrated after 0.025 Gy exposure at HDR, whereas LDR require higher doses (2 Gy) presumably through activation of adaptation mechanisms at LDR.

Low-dose IR of ApoE+/+ wild type and ApoE−/− mice revealed significant early changes of inflammatory plasma markers depending on the delivered dose rates and the inflammatory status of the subject. Starting at doses of 0.005 Gy, wild type mice showed stronger proinflammatory cytokine responses than ApoE−/− mice especially 7 and 28 days after IR.

Non-linear cytokine release and change of adhesion properties after low-dose IR were confirmed in vitro and indicate that ECs might contribute to the systemic effects measured by plasma markers.

The low-dose IR effects presented here may have important implications for people with chronic e.g., environmental exposures (LDR) or in advanced medical radio-diagnostics (HDR) and may improve our comprehension of differential IR responses at doses below 0.1 Gy.

## Figures and Tables

**Table 1:** RT-PCR Primers and Probes.

Gene/Product Size	Forward Primer 5′-3′	Probe 5′-3′	Reversed Primer 5′-3′
mCD68/73 bp NM_001291058	CTCAGCTGCCTGACAAGGGA	FAM-TCGGGCCATGTTTCTCTTGCAACCGT-BHQ1	AGAGGCAGCAAGAGGGACTG
mKi67/99 bp NM_001081117	AGCAGACGAGCAAGAGACAA	FAM.-CCCAGCACTCCAAAGAAACCCAC-BHQ1	TACAGGGAGAGTTTGCATGG
mB2m/91 bp [33] NM_009735	TGAGACTGATACATACGCCTGCA	HEX-ATGGCCGAGCCCAAGACCGTC-BHQ1	GATGCTTGATCACATGTCTCGATC
m18S/102 bp NR_003278	AGGAATTCCCAGTAAGTGCG	HEX-TCCCTGCCCTTTGTACACACCGCC-BHQ1	GCCTCACTAAACCATCCAA

## Data Availability

The data presented in this study are available on request from the corresponding author.

## Abbreviations

ApoE	Apolipoprotein E
CD68	Cluster of Differentiation 68
EC	endothelial cell
G-CSF	Granulocyte-Colony Stimulating Factor
INF	Interferon
IL	Interleukin
IR	irradiation
KC	Keratinocyte-derived chemokine
Ki67	Marker of proliferation Ki-67
LDR	low dose rate, HDR: high dose rate
MCP1	Monocyte Chemoattractant Protein 1
NO	nitric oxide
RANTES	Regulated on Activation Normal T cell Expressed and Secreted
ROS	reactive oxygen species
sE-selectin	soluble Endothelial Leukocyte Adhesion Molecule-1
sICAM	soluble Intercellular Adhesion Molecule 1
sVCAM	soluble Vascular Adhesion Molecule 1
TGFβ	Transforming Growth Factor beta 1
